# Stay-Green Trait Enhances Grain Yield, Nutritional Quality, and Seed Germination Ability in Oat (*Avena sativa* L.) on the Qinghai–Tibet Plateau

**DOI:** 10.3390/plants14162500

**Published:** 2025-08-12

**Authors:** Huimin Duan, Lingling Liu, Wenhu Wang, Sida Li, Zhenghai Shi, Guoling Liang, Wenhui Liu

**Affiliations:** Key Laboratory of Superior Forage Germplasm in the Qinghai-Tibetan Plateau, Qinghai Academy of Animal Science and Veterinary Medicine, Qinghai University, Xining 810016, China; dhm_80@163.com (H.D.); liu_ling_60@sina.com (L.L.); wwh01112021@163.com (W.W.); lisida_sir@163.com (S.L.); shizhenghaishiwo@gmail.com (Z.S.); qhliangguoling@163.com (G.L.)

**Keywords:** oat, stay-green, grain yield, nutritional quality, seed germination

## Abstract

Oat is a dual-purpose crop valued for both grain and forage. The stay-green (SG) trait, which delays leaf senescence and prolongs photosynthesis, has been shown to increase yield and quality in several crop species, yet its performance across diverse environments in oats remains underexplored. In this study, multi-location field trials were conducted in Ledu, Huangzhong and Haiyan, Qinghai Province, China, to comprehensively evaluate the performance of stay-green oat lines. The traits evaluated included grain yield components, nutritional quality, and seedling establishment traits. A TOPSIS (technique for order preference by similarity to an ideal solution) model, coefficient of variation (CV) and *G* × *E* (genotype × environment) visualization were used to assess adaptability, stability, and genotype × environment interactions. On average, the stay-green lines exhibited an 16.00% increase in plot yield and a 22.93% increase in thousand-grain weight compared to controls. Notable improvements were also observed in the starch (7.58% LN_SG in HZ and HY) and protein (3.58%, QY5_SG all the sites) contents, as well as multiple seedling establishment indices, with the seedling vigor indices increasing by more than 50%. Stability analysis further showed that the stay-green lines were stable in spike length, thousand-grain weight, water-soluble carbohydrates, and seed and seedling vigor. TOPSIS analysis identified ‘LN_SG’ as the top-performing and most adaptable genotype across all environments. Overall, stay-green oat lines demonstrated superior performance in grain yield, nutritional quality, and seedling establishment. These findings highlight their potential for field application and their value as parental materials in oat breeding programs enhancing environmental adaptability and stability.

## 1. Introduction

Oat is a versatile cereal crop valued for its rich nutritional composition and remarkable adaptability to impoverished environments. In high-altitude and cold arid agroecosystems, oat plays a crucial role not only in food and feed production but also in soil conservation, crop rotation, and integrated livestock systems [1,2,3]. However, the sustainability of oat production is increasingly challenged by the rising frequency of extreme weather events (e.g., drought, heat, and frost) driven by global climate change. These environmental pressures contribute to yield instability, but the market demand for stable grain quality and high seed vigor is growing.

To address these challenges, genetic improvement efforts have increasingly focused on traits that enhance stress resilience without compromising yield or quality. One such trait is “stay-green (SG),” which is characterized by delayed leaf senescence and sustained photosynthetic activity during the postanthesis period. Initially, identified in leguminous crops, the stay-green phenotype has gained prominence in cereal breeding due to its associations with extended grain filling, enhanced radiation use efficiency (RUE), and improved yield stability under abiotic stress [4,5,6,7,8,9,10,11]. In wheat, for example, stay-green genotypes have demonstrated the capacity to maintain higher chlorophyll contents, net photosynthetic rates, and antioxidant enzyme activities under heat stress, thereby supporting more effective grain development [6,12,13,14]. Similarly, in maize and soybean, stay-green lines exhibit improved nitrogen use efficiency and more efficient biomass partitioning, both of which contribute to higher yields [15,16]. In addition, in maize, the reduced remobilization of dry matter in stay-green lines also contributes to improved stem integrity, thereby reducing the risk of lodging [17].

Notably, in oats, stay-green genotypes are able to maintain green foliage even when the grains have reached physiological maturity, whereas traditional cultivars typically exhibit complete senescence of leaves and stems at this stage. This prolonged functional activity of vegetative organs during grain maturation significantly enhances the forage value of oat plants at harvest. However, despite the well-documented advantages of the stay-green trait in other cereal crops, its breeding potential and application value in oats remain largely unexplored. To date, only limited research has reported a negative correlation between the onset of leaf senescence at anthesis and the concentration of water-soluble carbohydrates in oat plants [18]. The lack of comprehensive studies on stay-green expression in oat under diverse environmental conditions hampers the identification of its agronomic potential, particularly regarding grain biomass accumulation, nutritional composition, and seed vigor.

Grain quality in oat is a multifaceted trait that encompasses both nutritional value and germination capacity. Oat grains are notable for their high protein content (12%~20%) and a balanced amino acid profile, including a relatively high lysine concentration (~4.5 g per 100 g protein) [19]. The lipid fraction contains functional components such as linoleic acid and tocopherols [20], whereas the starch content is relatively low but characterized by a higher proportion of amylose and finer starch granules, resulting in increased viscosity and water-holding capacity [21]. Concurrently, germination-related traits—such as germination energy, germination index, and seedling vigor index—are essential for successful field emergence and early seedling establishment [22]. These traits are strongly influenced by genotype–environment (*G* × *E*) interactions [23]. For example, drought stress may elevate protein concentration at the expense of starch biosynthesis, ultimately reducing seed vigor and impairing seedling performance [24]. This complexity underscores the importance of evaluating oat grain traits under diverse environmental conditions to elucidate the interplay between stay-green expression and seed quality.

The northeastern Qinghai–Tibet Plateau presents a natural environmental gradient that is ideally suited for investigating genotype–environment interactions. The region encompasses three ecologically distinct zones—Ledu (warm-temperate semi-arid), Huangzhong (subalpine semi-humid), and Haiyan (high-altitude cold arid agro-pastoral ecotone)—each with unique production objectives and prevailing abiotic stress factors. Ledu is prone to intermittent drought during the grain-filling period; Huangzhong experiences cold stress and a shortened growing season; and Haiyan is characterized by intense solar radiation, frost, and extreme subzero temperatures. These contrasting environmental conditions provide an optimal context for examining the relationship between stay-green expression and key grain traits under abiotic stress.

In this study, three pairs of oat lines with similar genetic backgrounds (LN_CK/LN_SG, QY3_CK/QY3_SG, QY5_CK/QY5_SG) were used. The stay-green lines were derived through long-term field selection based on phenotypic performance from their respective backgrounds, which allows a more precise assessment of the stay-green phenotype under different environments. Given the existing knowledge gap concerning the influence of the stay-green trait on oat grain yield components, nutritional quality, and seed vigor under varying environmental conditions, this study aimed to assess the agronomic performance of stay-green oat lines across three ecologically distinct zones in Qinghai Province. By integrating multi-environment field trials with comprehensive phenotypic analyses, we aimed to elucidate the functional significance of the stay-green trait in oat and to provide both theoretical insights and practical recommendations for its application in breeding programs targeting high-quality oat production.

## 2. Results

### 2.1. Yield Performance in Different Ecological Regions

Spike length and fertile spikelet number were significantly affected by environmental conditions, genotype, group (CK/SG) and their interactions. The longest average spike length was recorded in LD (24.60 mm), while the highest fertile spikelet number occurred in HZ (52.44). The stay-green (SG) group had consistently enhanced spike length across environments, with increases of 8.77% in LD and 12.89% in HY. Among genotypes, QY3 and QY5 exhibited the most pronounced responses to SG. In LD, the spike length of QY3_SG reached 31.19 mm, representing an 11.51% increase relative to the control (CK), whereas QY5_SG in HY achieved 26.24 mm, reflecting a 25.43% increase. In contrast, LN_SG showed no significant difference from CK. Among the control groups, QY3_CK exhibited greater spike length in LD, while QY5_CK performed better in HZ. In SG lines, QY5_SG consistently maintained longer spike length in HZ.

The SG group also had increased fertile spikelet number across all locations, with improvements ranging from 6.12% in HY to 33.54% in HZ. QY3_SG showed the greatest improvement, with increases of 34.97% in HY and 59.69% in LD. LN_SG exhibited significant improvements in LD (+29.48%) and HZ (+63.73%). Among the CK materials, QY3_CK exhibited the highest fertile spikelet number in LD, QY5_CK performed best in HZ, and LN_CK led in HY. Among the SG materials, QY3_SG consistently outperformed other genotypes across all environments, indicating that SG further amplified genotypic differences.

Single-plant grain yield was significantly affected by environment, genotype, group (CK/SG) and their interactions, with the highest average yield recorded in HZ (5.04 g), followed by LD (3.67 g) and HY (2.78 g). The SG group had consistently improved single-plant yield across all locations, with increases ranging from 6.59% in HY to 34.40% in HZ. Among the genotypes, QY3_SG showed the greatest increase, with yields rising by 38.89% in HZ and 38.39% in LD. LN_SG also exhibited significant gains in LD (+30.78%) and HZ (+57.91%), whereas QY5_SG showed a significant increase only in HZ (+14.94%). Among the CK materials, QY5_CK achieved its highest single-plant yield in HZ, whereas QY3_CK performed better overall in HZ. Among the SG materials, QY3_SG consistently outperformed other genotypes across all environments, indicating that SG further amplified the yield advantage of superior genotypes, particularly in HZ and HY.

Plot yield exhibited a similar pattern of variation, with HZ recording the highest average yield (2743.71 g), significantly surpassing LD (1892.90 g) and HY (1750.31 g). SG improved plot yield across all locations, with increases ranging from 9.21% in HY to 40.50% in LD. LN_SG exhibited significant improvements across all locations, particularly in LD (+69.62%) and HZ (+34.43%). QY3_SG also showed improvements across all locations, particularly in HZ (+6.75%) and LD (+23.17%). QY5_SG exhibited improvements in LD (+31.45%) and HY (+9.19%). Among the CK materials, QY3_CK recorded the highest plot yield. Among the SG materials, QY3_SG maintained an advantage in HZ and HY, whereas LN_SG outperformed other genotypes in LD.

**Figure 1:**
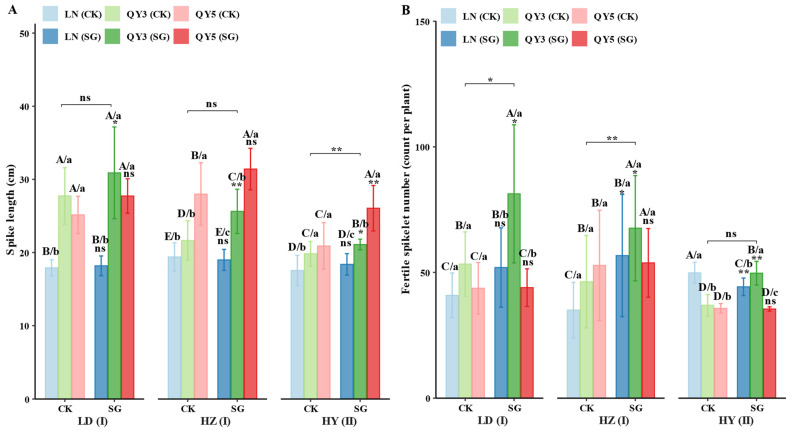

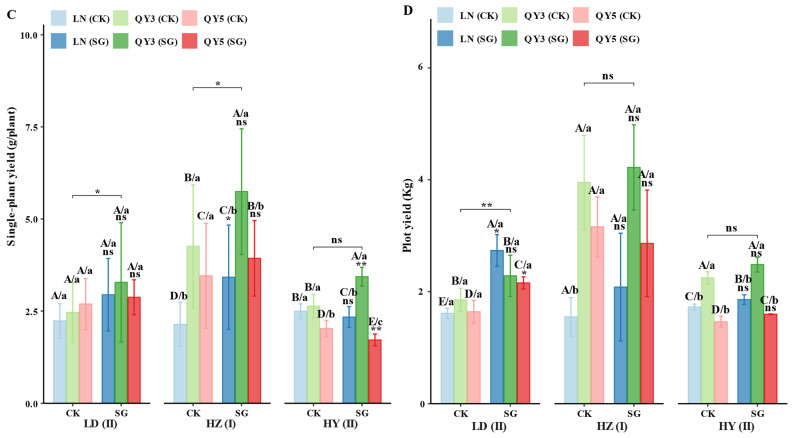
Effects of ecological region, genotype, and stay-green (SG) group on oat yield-related traits. Panels (**A**–**D**) represent the following traits across locations (LD, HZ, HY), genotypes (LN, QY 3, QY 5), and group (CK/SG): (**A**) spike length; (**B**) fertile spikelet number; (**C**) single-plant yield; (**D**) plot yield. Statistical significance symbols are based on Games–Howell test results: lowercase letters indicate differences among genotypes within the same location and group (CK/SG) (*p* < 0.05); uppercase letters indicate differences among genotype × group (CK/SG) combinations within the same location (*p* < 0.05); ns, *, and ** indicate non-significant, significant (*p* < 0.05), and highly significant (*p* < 0.01) differences between CK and SG within the same genotype and location, respectively; significance bridges indicate overall differences between CK group and SG group; Roman numerals indicate differences between experimental locations. All letters are ranked by group means from high to low. Error bars represent ± standard error (SE).

In summary, the SG group consistently improved yield-related traits, including spike length, fertile spikelet number, single-plant yield, and plot yield across all locations. The increase in spike length ranged from 8.77% to 12.89%, fertile spikelet number from 6.12% to 33.54%, single-plant yield from 6.59% to 34.40%, and plot yield from 9.21% to 40.50%, although the specific gains varied among genotypes and environments. QY3_SG exhibited a clear advantage in yield-related traits, particularly in HZ and HY. LN_SG exhibited significant increases in both plot yield and single-plant yield at the LD site (Figure 1).

### 2.2. Grain Phenotypic Traits

Grain length, width, and thousand-grain weight (TGW) were significantly affected by environment, group (CK/SG), genotype, and their interactions, with optimal traits and materials varying among locations. HZ exhibited the greatest grain length (13.58 mm) and width (2.97 mm), significantly surpassing LD and HY, whereas HY recorded the highest TGW (38.36 g), significantly exceeding both HZ and LD. The effect of the SG group on grain morphology was location-dependent; SG increased grain length by 4.13% in HZ, but had negligible effects in LD (+0.21%) and a slight decrease in HY (0.84%). Grain length in QY3_SG and QY5_SG showed positive responses in HZ, increasing by 10.88% and 3.22% compared with QY3_CK and QY5_CK, respectively, with no significant effects observed in LD or HY. Among the CK materials, QY3_CK exhibited the greatest performance in LD, LN_CK performed best in HZ, and QY5_CK performed best in HY. Among the SG materials, QY3_SG performed well in HZ, whereas QY5_SG maintained an advantage in HY.

For grain width, SG increased values by 4.26% in LD but had negligible effects in HZ (0.17%) and HY (0.85%). In LD, all SG lines—LN_SG, QY3_SG, and QY5_SG—showed improved performance, particularly QY3_SG, which exhibited the highest increase (+6.66%). In HZ, only QY5_SG exhibited a slight increase (+2.31%), whereas LN_SG and QY3_SG remained unchanged. No significant increases were observed in HY. Across all environments, QY3 exhibited the greatest grain width under both CK and SG.

The SG group also significantly increased thousand-grain weight (TGW) across all locations, with increases ranging from 16.5% in HY to 29.68% in HZ. LN_SG exhibited the greatest increase in HZ (+93.47%), whereas QY5_SG demonstrated improvements across all locations (ranging from +9.28% to +41.17%). QY3_SG showed significant increases in HZ (+11.9%) and HY (+13.5%), but exhibited no significant change in LD. Among the CK materials, QY5_CK recorded the highest TGW in LD and HZ, whereas QY3_CK performed better in HY. Among the SG materials, LN_SG, QY5_SG, and QY3_SG each exhibited location-specific advantages, demonstrating a strong genotype × environment × group (CK/SG) interaction affecting grain traits.

**Figure 2:**
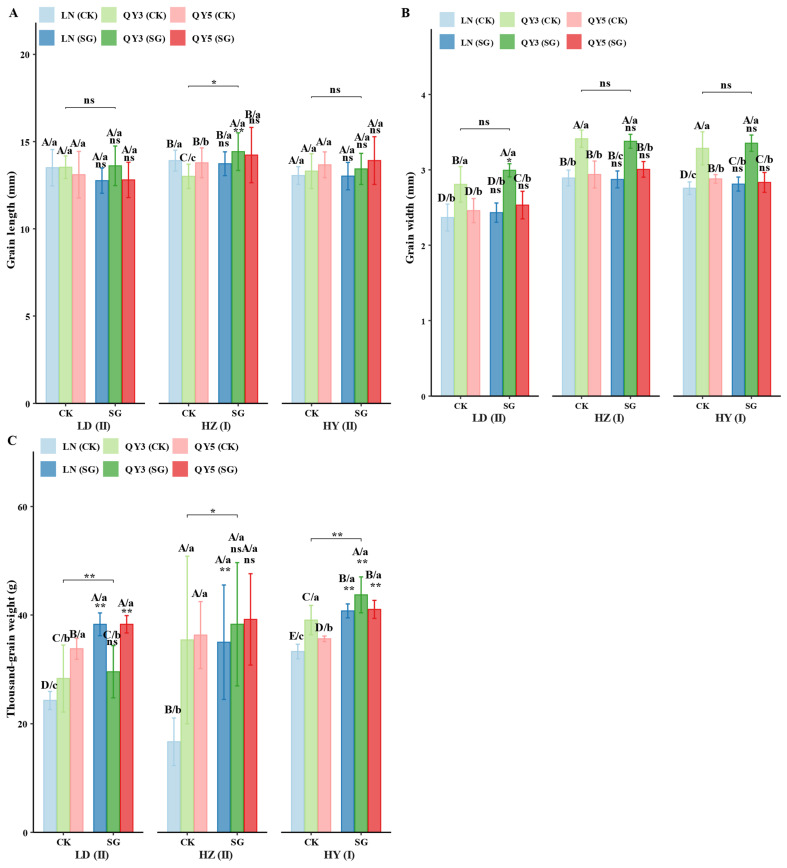
Regulatory effects of ecological region, genotype, and stay-green (SG) group on oat grain morphology and thousand-grain weight. Panels (**A**–**C**) represent (**A**) grain length; (**B**) grain width; and (**C**) thousand-grain weight. Statistical significance symbols are based on Games–Howell test results: lowercase letters indicate differences among genotypes within the same location and group (CK/SG) (*p* < 0.05); uppercase letters indicate differences among genotype × group (CK/SG) combinations within the same location (*p* < 0.05); ns, *, and ** indicate non-significant, significant (*p* < 0.05), and highly significant (*p* < 0.01) differences between CK and SG within the same genotype and location, respectively; significance bridges indicate overall differences between CK group and SG group; Roman numerals indicate differences between experimental locations. All letters are ranked by group means from high to low. Error bars represent ± standard error (SE).

In summary, the SG group significantly improved grain length, grain width, and thousand-grain weight (TGW), with the greatest impact observed on TGW. Grain length significantly increased in HZ (+4.13%), whereas effects in LD and HY were minimal or slightly negative. Grain width significantly increased in LD (+4.26%), but showed no significant positive effects in HZ and HY. TGW consistently increased across all locations, with increases ranging from 16.5% in HY to 29.68% in HZ. Among genotypes, LN_SG exhibited the greatest increase in thousand-grain weight, reaching 93.47% in HZ. QY3_SG and QY5_SG responded positively to the SG, although the degree of response varied by location (Figure 2).

### 2.3. Grain Nutrient Composition

Grain starch, water-soluble carbohydrate (WSC), protein, and fat contents were significantly influenced by environment, with responses in the SG group dependent on genotype and location. HZ provided the most favorable conditions for starch accumulation (25.71%) and WSC content (21.65%), whereas LD favored protein accumulation (13.61%). Both HZ and HY exhibited higher fat contents, averaging 4.85%. SG materials increased grain starch content in HZ (+11.88%) and HY (+3.40%), but decreased it in LD (−4.07%). Starch content in QY3_SG increased at all locations (+2.01% to +2.94%), whereas LN_SG exhibited significant increases in LD (+7.46%) and HY (+7.69%). Among the SG materials, QY3_SG performed better in LD and HY, whereas LN_SG performed best in HZ.

The SG group increased grain WSC content in HY (+6.84%), but caused slight decreases in LD and HZ (−0.29% and −1.79%). QY3_SG showed consistent increases in HZ (+1.74%) and HY (+7.04%), whereas LN_SG and QY5_SG exhibited strong location-dependent responses. Notably, QY5_SG exhibited a significant increase in HY (+17.78%) but decreased in LD and HZ. Across all locations, both CK and the SG group of QY3 maintained the highest WSC content.

The response of protein content in the SG group was similar to those of starch and WSC. SG increased protein content in oat grains in HZ (+4.47%) and HY (+1.37%), but had no significant effect in LD. QY5_SG exhibited continuous increases in protein content across all locations, with a significant increase in LD (+4.79%). Protein content of LN_SG sharply increased in HZ (+27.13%) but decreased in LD (−6.12%), whereas QY3_SG showed an increase only in HY (+2.62%). Among the SG materials, QY5_SG performed best across all locations.

Fat content was higher in LD and HY, at 13.61% and 10.36%, respectively. The SG group had increased fat accumulation in LD (+1.14%) and HY (+2.46%), but was decreased in HZ (−1.87%). Fat content of QY3_SG increased in LD (+3.19%) and HZ (+1.63%), while LN_SG had increased fat content in HY (+3.08%) but was sharply decreased in HZ (−5.28%). QY5_SG showed an increase in HY (+3.69%) but decreased levels in LD and HZ. Across all environments, both the CK and SG groups of LN exhibited the highest fat content.

**Figure 3:**
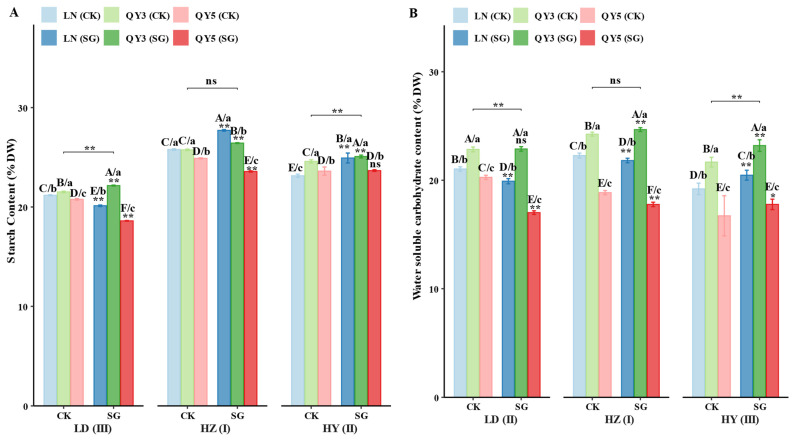

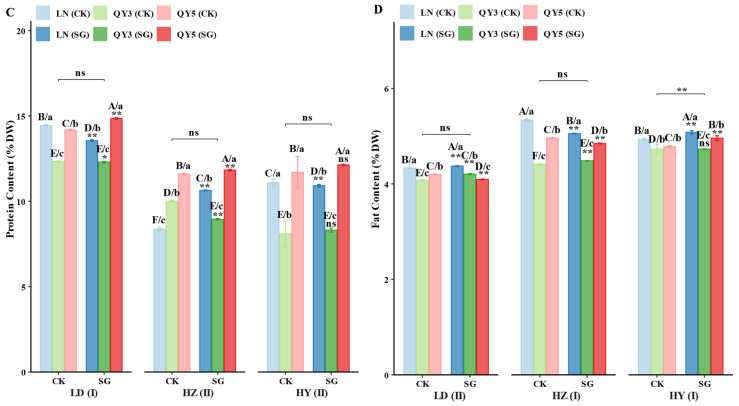
Effects of ecological region, genotype, and stay-green (SG) group on oat grain quality traits. Panels (**A**–**D**) represent (**A**) starch content; (**B**) water-soluble carbohydrates (WSC) content; (**C**) protein content; and (**D**) fat content. Statistical significance symbols are based on Games–Howell test results: lowercase letters indicate differences among genotypes within the same location and group (CK/SG) (*p* < 0.05); uppercase letters indicate differences among genotype × group (CK/SG) combinations within the same location (*p* < 0.05); ns, *, and ** indicate non-significant, significant (*p* < 0.05), and highly significant (*p* < 0.01) differences between CK and SG within the same genotype and location, respectively; significance bridges indicate overall differences between CK group and SG group; Roman numerals indicate differences between experimental locations. All letters are ranked by group means from high to low. Error bars represent ± standard error (SE).

In summary, environmental factors are the primary drivers of variation in starch, water-soluble carbohydrate (WSC), protein, and fat contents, whereas the effects of the stay-green (SG) group on these nutritional traits depend on genotype and location. SG enhanced starch accumulation in HZ and HY, particularly benefiting the LN genotype. SG increased WSC content only in HY, with QY3 consistently maintaining high WSC levels across all environments and responding positively to SG in HZ and HY. SG increased protein content in HZ and HY, with QY5_SG consistently exhibiting the highest protein levels across all locations. SG also increased fat content in LD and HY, with LN maintaining high fat accumulation across all environments and further benefiting from SG in these locations (Figure 3).

### 2.4. Seed Germination and Seedling Establishment

Seed germination-related indicators, including germination energy, germination percentage, germination index, and seedling percentage, were significantly influenced by environmental factors, group (CK/SG) genotypes, and their interactions. Among the locations, HZ exhibited the highest values for germination energy (87.51), germination percentage (90.11%), germination index (63.46), and seedling percentage (89.39%), significantly surpassing LD and HY. The SG group showed generally improved seed germination indicators; however, genotype responses varied. QY3_SG showed a consistent positive effect on germination energy across all locations. QY5_SG exhibited significant improvements in germination percentage and related traits in LD and HZ. LN_SG performed best in HY, particularly for germination percentage and seedling percentage. These results indicate that the SG group had broadly enhanced seed germination and early seedling establishment, although its effects are modulated by genotype × environment interactions.

The seed vigor index and seedling vigor index are key indicators for assessing seed and early seedling quality, and are significantly influenced by location, group (CK/SG), genotype, and their interactions. HZ and LD exhibited the highest values (HZ: seed vigor index 13.27 and seedling vigor index 17.33; LD: 11.59 and 20.65), significantly outperforming HY. The SG group improved seed and seedling vigor indices across all genotypes and locations, with LN_SG exhibiting the greatest improvement. In HY, LN_SG increased seed vigor index and seedling vigor index by as much as 148.84% and 330.71%, respectively. Among the SG materials, QY3_SG performed best in LD, LN_SG in HZ, and QY5_SG in HY, highlighting the importance of genotype–environment matching for maximizing vigor improvement.

Seedling growth traits, typically comprising seedling height, seedling weight, root length, and root weight, were highest in LD and HZ. LD favored seedling height and root traits, whereas HZ exhibited the highest seedling weight (average 85.75 mg). The SG group significantly improved growth parameters across all locations, with improvements ranging from approximately 4.44% to over 73.38%, depending on the trait and location. LN_SG showed significant increases, particularly in HY, where seedling height and root length increased by 207.03% and 172.53%, respectively. QY3_SG exhibited significant improvements at all locations, whereas the responses of various traits in QY5_SG varied, including a reduction in root weight in HZ. These findings indicate that the SG group has enhanced early biomass accumulation and root development in seedlings, which supports improved seedling vigor and potential stress tolerance, with effects modulated by genotype and environment.

**Figure 4:**
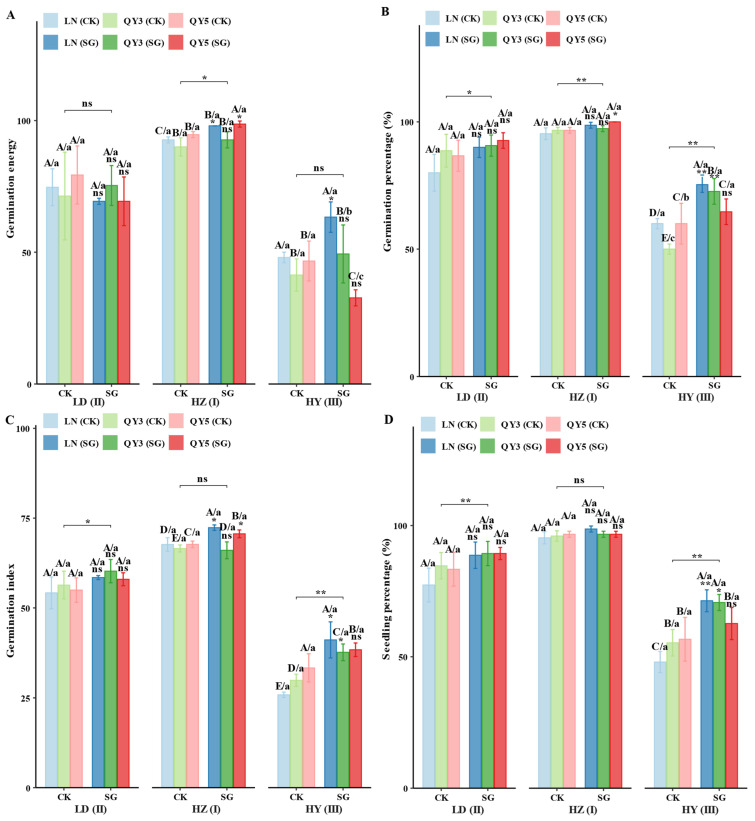

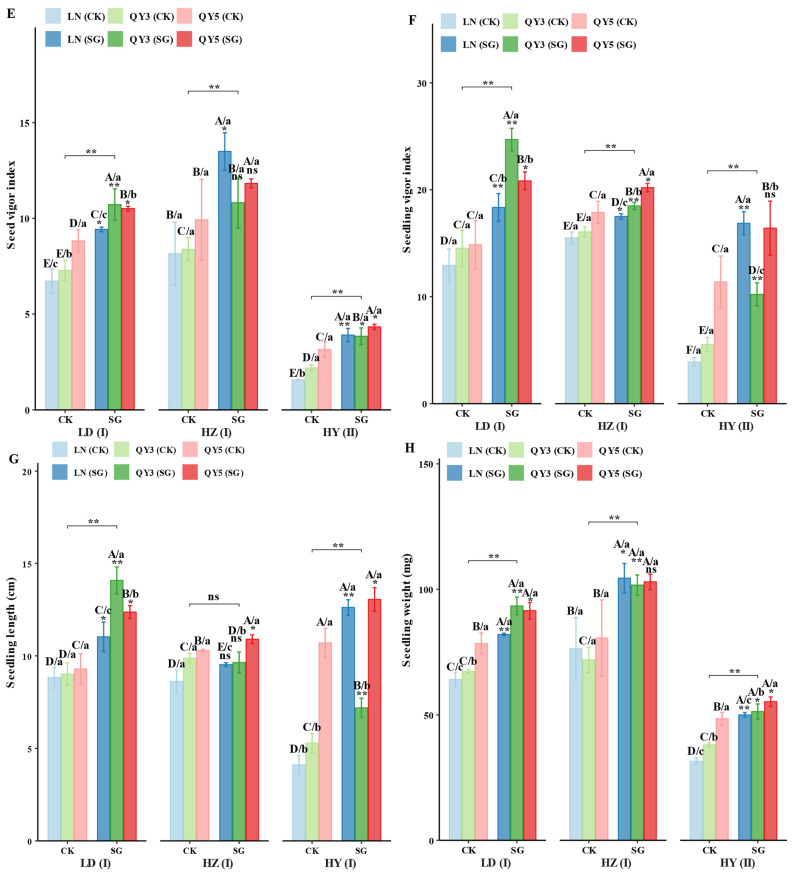

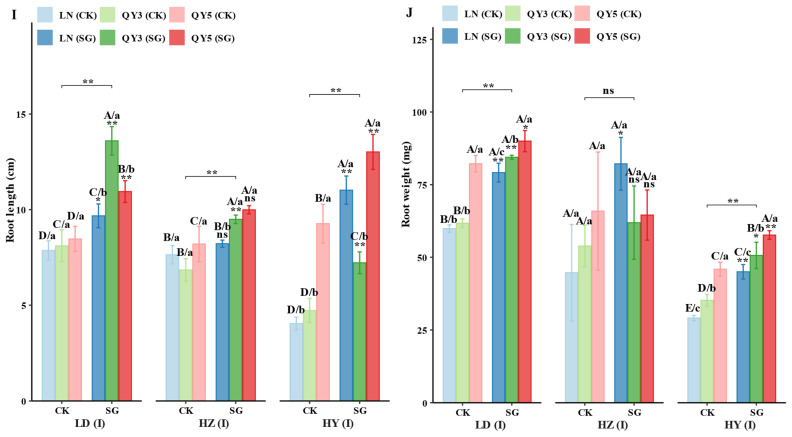
Effects of ecological region, genotype, and stay-green (SG) group on oat grain germination performance. Panels (**A**–**J**) represent (**A**) germination energy; (**B**) germination percentage; (**C**) germination index; (**D**) seedling percentage; (**E**) seed vigor index; (**F**) seedling vigor index; (**G**) seedling length; (**H**) seedling weight; (**I**) root length; and (**J**) root weight. Statistical significance symbols are based on Games–Howell test results: lowercase letters indicate differences among genotypes within the same location and group (CK/SG) (*p* < 0.05); uppercase letters indicate differences among genotype × group (CK/SG) combinations within the same location (*p* < 0.05); ns, *, and ** indicate non-significant, significant (*p* < 0.05), and highly significant (*p* < 0.01) differences between CK and SG within the same genotype and location, respectively; significance bridges indicate overall differences between CK group and SG group; Roman numerals indicate differences between experimental locations. All letters are ranked by group means from high to low. Error bars represent ± standard error (SE).

In summary, in the stay-green (SG) group, most seed germination and seedling vigor traits were consistently and significantly enhanced, although the magnitude of the effects varied with genotype and environment. The effect of SG on germination energy demonstrates a strong genotype × environment interaction, with QY3_SG uniquely exhibiting a stable positive increase across all locations. For germination percentage, germination index, and seedling percentage, SG broadly enhances the performance of multiple genotypes. QY5_SG performs well in LD and HZ, whereas LN_SG exhibits a particular advantage in HY, highlighting genotype × environment interactions. SG also significantly increases seed vigor and seedling vigor indices across all genotypes and environments, with LN_SG exhibiting the greatest improvement in HY. Regarding seedling growth traits (length, weight, root length, root weight), SG enhances biomass accumulation and root development, thereby promoting early growth and stress tolerance. LN_SG exhibits the most pronounced positive response, particularly in HY. Overall, SG broadly enhances seed vigor and seedling establishment; however, the response is dependent on genotype × environment interactions. QY3_SG and LN_SG consistently exhibit stable and significant positive traits across multiple environments, making them priority breeding materials for leveraging the benefits of SG (Figure 4).

### 2.5. Stability and Adaptability of Grain Yield, Nutritional Quality, and Germination Traits Under Genotype × Group (CK/SG) Interaction in Oat

Multi-trait stability analysis revealed distinct performance patterns among genotypes. The LN genotype exhibited trait-specific stability, with LN–CK showing high stability in plot yield, whereas LN–SG demonstrated superior stability in seed vigor and germination-related traits. In contrast, QY3 exhibited marked instability in traits such as spike length (coefficient of variation [CV] up to 30.2%), number of fertile spikelets per plant (CV up to 46.1%), single-plant yield, plot yield, and protein content, indicating high sensitivity to environmental fluctuations and resulting in considerable performance variability. By comparison, QY5 exhibited the highest overall stability, with QY5–CK demonstrating superior stability in key yield and quality traits, including spike length (CV = 9.5%), single-plant yield (CV = 25.8%), thousand-grain weight (CV = 4.8%), and protein content (CV = 11.5%). Additionally, QY5–SG exhibited excellent stability in water-soluble carbohydrate (WSC) content (CV as low as 2.5%) and in most seed and seedling vigor traits, including root length, seedling height, and vigor index.

Each genotype–group (CK/SG) combination exhibited distinct patterns of adaptability and yield potential, underscoring the critical role of environmental effects. LN–SG exhibited the highest thousand-grain weight (40.67 g in HY) and the highest starch content in HZ (27.71%). QY3–SG recorded the highest number of fertile spikelets per plant (67.64), single-plant yield (5.82 g), and plot yield (4219.74 g) in HZ. However, this superior productivity is highly dependent on the favorable conditions of the HZ environment, reflecting QY3’s characteristic high-yield yet unstable performance pattern. In contrast, QY5 is recognized as a high-quality and broadly adaptable genotype, demonstrating stable performance across multiple environments, alongside excellent yield and quality traits. For instance, QY5–SG exhibited outstanding spike length in HZ (31.14 mm), achieved the highest protein content in LD (14.91% DW), and demonstrated favorable seed and seedling vigor traits, including root length (12.37 mm) and seedling height (10.94 mm).

Analysis of variance revealed that location, genotype, group (CK/SG), and their interactions exerted moderate effects on the comprehensive quality of oat grains; however, the influence of each factor varied significantly across individual traits. Yield-related and grain traits were primarily influenced by single factors and two-factor interactions among location, genotype, and group (CK/SG), whereas the three-factor interaction was not significant. Nutrient composition indices were significantly affected by single factors, two-factor interactions, and the three-factor interaction. Germination-related indicators were less influenced by genotype, but were significantly affected by the single factors of group (CK/SG) and location, as well as two-factor and three-factor interactions.

**Figure 5:**
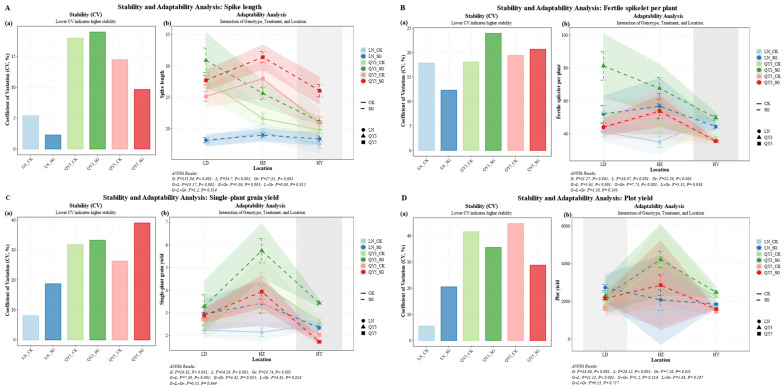

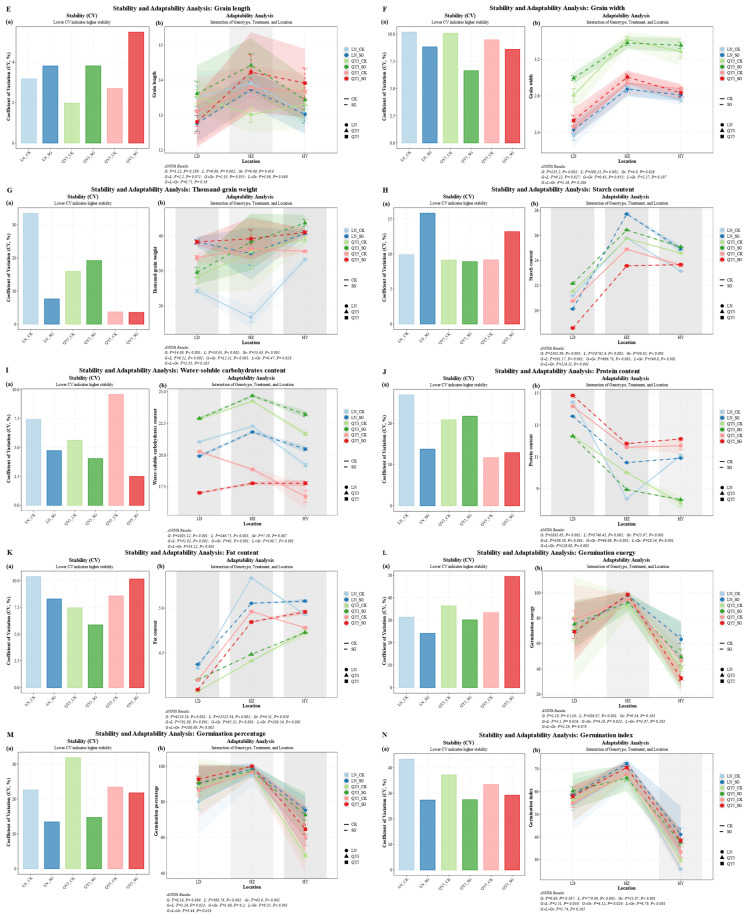

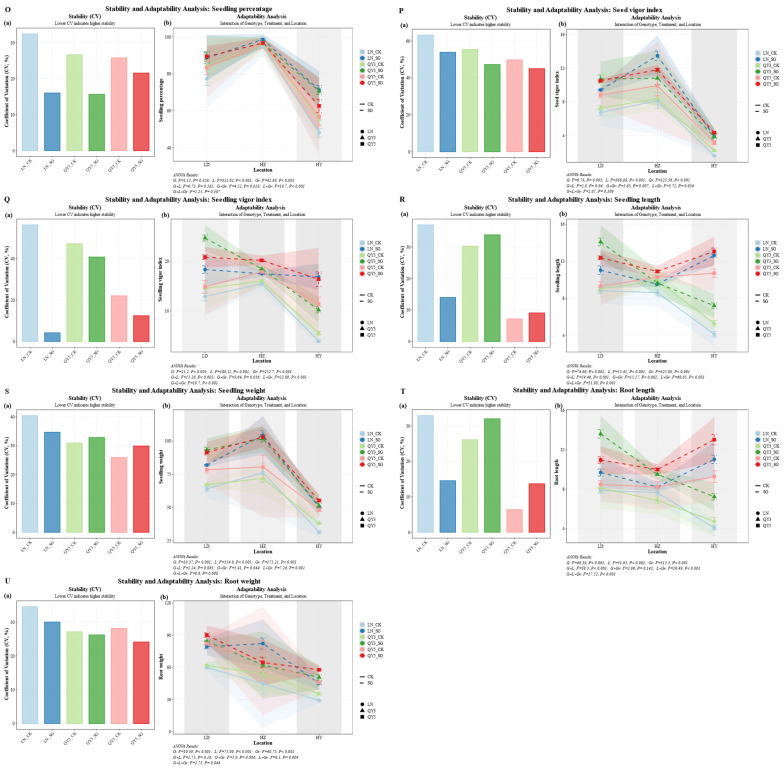
Stability and adaptability analysis of oat yield, nutritional quality, and germination-related traits under genotype and group (CK/SG) combinations. Panels (**A**–**U**) represent (**A**) spike length; (**B**) fertile spikelet number; (**C**) single-plant yield; (**D**) plot yield; (**E**) grain length; (**F**) grain width; (**G**) thousand-grain weight; (**H**) starch content; (**I**) water-soluble carbohydrate content; (**J**) protein content; (**K**) fat content; (**L**) germination energy; (**M**) germination percentage; (**N**) germination index; (**O**) seedling percentage; (**P**) seed vigor index; (**Q**) seedling vigor index; (**R**) seedling length; (**S**) seedling weight; (**T**) root length; and (**U**) root weight. The (**a**) subpanels show the coefficient of variation (CV), where lower CV values indicate higher stability across environments. The (**b**) subpanels present adaptability analysis; greater curve crossover indicates stronger genotype, group (CK/SG), and environment interactions, whereas more parallel trends reflect weaker interactions. In the analysis of variance shown in the figure, capital letter G represents genotype (LN, QY3, and QY5), L represents location (LD, HZ, and HY), and Gr denotes the grouping factor (CK and SG).

In summary, with respect to environmental effects, HZ was identified as the optimal site for maximizing yield and carbohydrate accumulation, whereas LD favored higher protein accumulation. Regarding genotype selection, LN_SG demonstrated potential for enhanced thousand-grain weight and starch accumulation. QY3_SG achieved maximum yield potential under the favorable conditions of HZ. For balancing yield, quality (particularly protein content), and stability, QY5 emerged as the most suitable genotype. Regarding phenotype-specific advantages, LN excelled in weight and quality-related traits, and QY3 performed well in yield-related traits, while QY5 demonstrated superiority in quality traits and plant vigor. The SG group exerted significant effects on all indicators except grain length. Notably, the group (CK/SG) effects on seed and seedling vigor (*F* = 123.36 and 252.7), seedling length (*F* = 325.09), seedling weight (*F* = 171.21), and root length (*F* = 315.3) were pronounced (Figure 5).

### 2.6. Comprehensive Evaluation of Adaptability and Stability Based on the AMMI Model

To further evaluate the adaptability and stability of genotype combinations across multiple environments, the additive main effects and multiplicative interaction (AMMI) model was employed. Building upon the preliminary assessment based on the coefficient of variation (CV) and interaction plots, this analysis treated genotype combinations as analytical units and experimental sites as environmental variables. AMMI1 biplots were constructed to visualize the overall response patterns of each combination across environments. The analysis was categorized by trait groups: yield and yield components, grain quality traits, and seed germination and seedling vigor traits.

**Figure 6:**
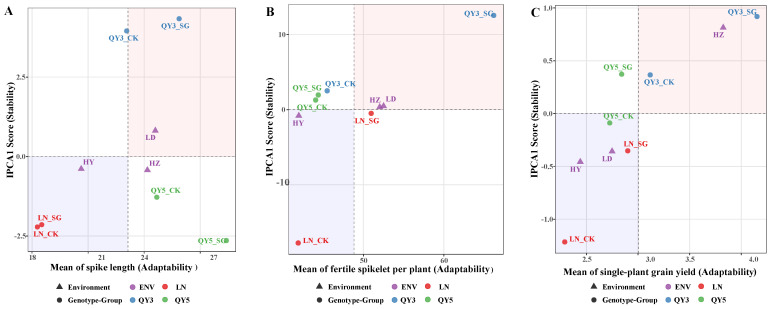

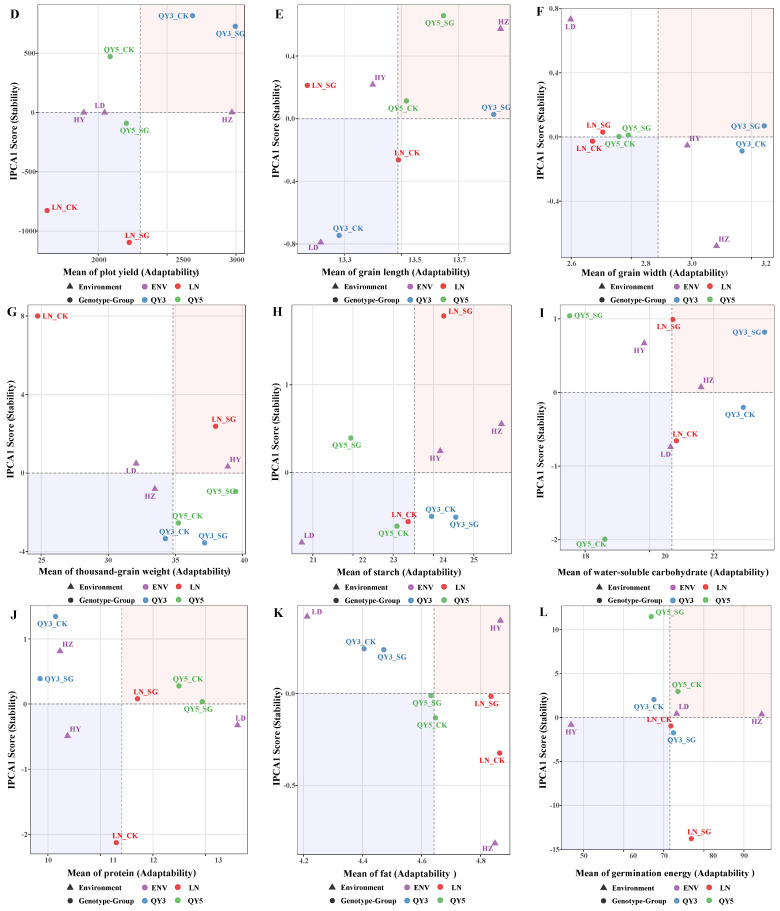

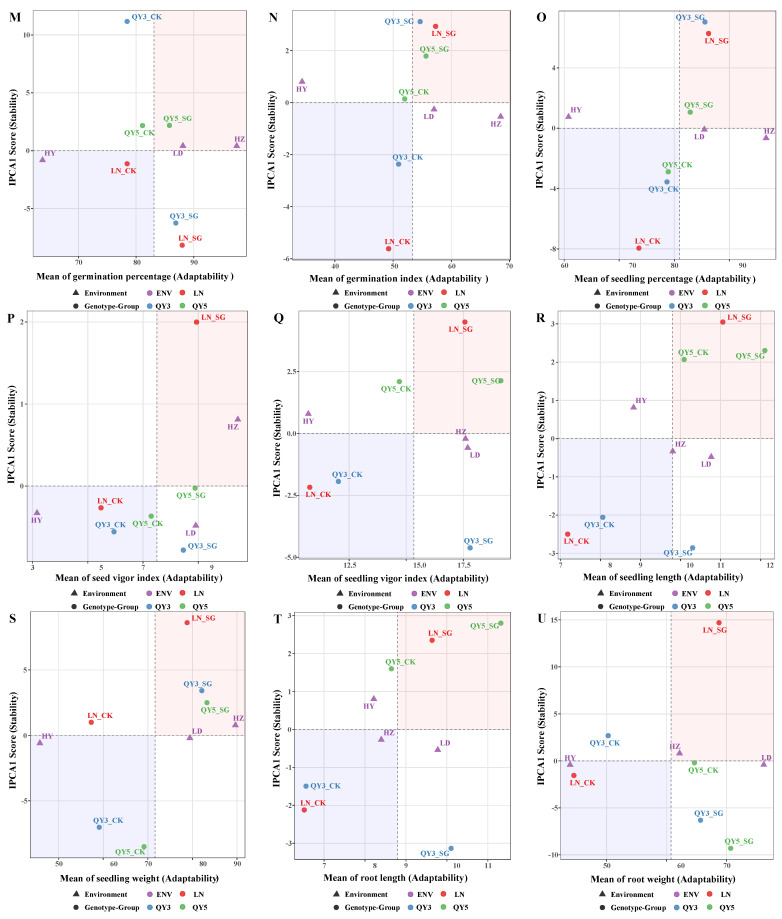
AMMI1 biplots for adaptability and stability of oat yield, nutritional quality, and germination-related traits. Panels (**A**–**U**) represent (**A**) spike length; (**B**) fertile spikelet number; (**C**) single-plant yield; (**D**) plot yield; (**E**) grain length; (**F**) grain width; (**G**) thousand-grain weight; (**H**) starch content; (**I**) water-soluble carbohydrate content; (**J**) protein content; (**K**) fat content; (**L**) germination energy; (**M**) germination percentage; (**N**) germination index; (**O**) seedling percentage; (**P**) seed vigor index; (**Q**) seedling vigor index; (**R**) seedling length; (**S**) seedling weight; (**T**) root length; and (**U**) root weight. These AMMI-based biplots illustrate the comprehensive evaluation of genotype–group combinations (depicted as circles) across various environments (depicted as triangles). The x-axis (mean performance) indicates adaptability, with higher values toward the right signifying greater adaptability. The y-axis (IPCA1 score) reflects stability, where smaller absolute values correspond to higher stability. The plot is divided into four quadrants by the grand mean line (vertical dashed line) and the IPCA1 zero line (horizontal dashed line). The right-side region (shaded in light red) denotes above-average performance (high adaptability), whereas the left-side region (shaded in light blue) indicates below-average performance (low adaptability). Circles in different colors represent different original genotypes.

For yield and its components, QY3_SG exhibited the highest single-plant yield, occupying the farthest position along the X-axis, indicating high yield potential and broad adaptability. In contrast, QY5_SG had the smallest absolute IPCA1 score, suggesting the highest yield stability (Figure 6C). In the case of plot yield, HZ-QY3_SG demonstrated the best adaptability (Figure 6D), but its distant position from the IPCA1 origin implied moderate stability. Although QY5_SG had a lower mean yield than QY3_SG, its IPCA1 score was closest to zero, indicating good environmental stability. For thousand-grain weight, the HZ and HY environments showed opposite interaction directions, being positioned at opposite ends of the IPCA1 axis. QY5_SG again showed both high adaptability and stability (Figure 6F).

Regarding grain quality traits, QY3_SG exhibited superior performance in starch and water-soluble carbohydrate (WSC) content (Figure 6H,I), while QY5_SG and LN_SG showed the greatest stability in protein and fat content (Figure 6J,K).

Traits related to seed germination and seedling vigor further revealed differential early-stage responses among combinations. For germination energy and percentage, LN_SG had the highest adaptability but relatively low stability, indicating sensitivity to environmental variation (Figure 6L,M). In contrast, LN_CK maintained moderate germination performance with the smallest absolute IPCA1 score, demonstrating consistent performance across environments. For the seed vigor index, QY5_SG combined high adaptability and high stability (Figure 6P). For seedling vigor index, SG genotypes generally showed higher adaptability but lower stability compared to CK lines (Figure 6Q). Overall, SG genotypes tended to have enhanced early vigor but showed increased sensitivity to environmental variation.

### 2.7. Comprehensive Performance Evaluation

**Figure 7:**
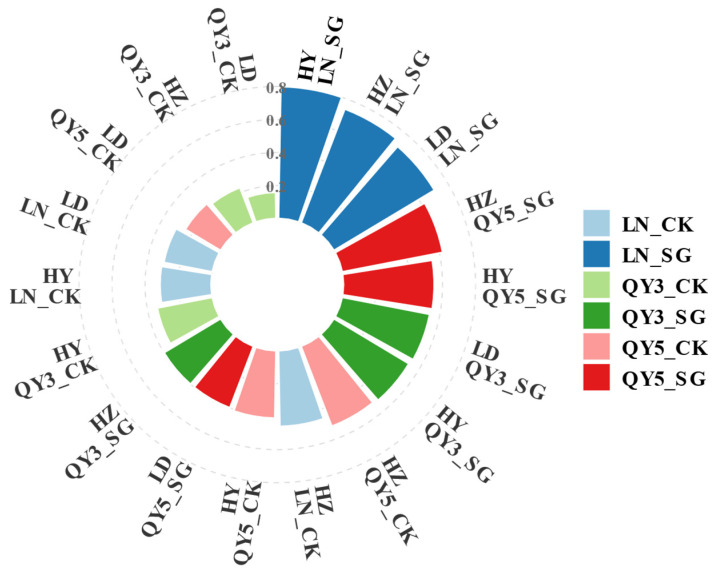
Comprehensive evaluation of different oat varieties and lines across multiple environments based on the TOPSIS model.

To further integrate and clarify genotype performance across environments, the TOPSIS method was employed as a complementary analysis (Figure 7). A comprehensive evaluation of oat genotypes across three environments was performed using the Technique for Order Preference by Similarity to Ideal Solution (TOPSIS) model (Figure 5). The stay-green genotype LN_SG consistently ranked first across all three locations, with relative proximity values of 0.70, 0.73, and 0.79 in LD, HZ, and HY, respectively. Notable regional differences were observed: in LD and HY, the top three genotypes were all stay-green types, whereas in HZ, only LN_SG and QY3_SG occupied the top two positions.

## 3. Discussion

### 3.1. Biomass Advantage of Stay-Green Oat Lines

The yield advantage of stay-green (SG) oat lines across multiple environments may result from maintaining a more efficient source–sink relationship during critical reproductive periods, thereby promoting greater biomass accumulation and enhancing the allocation of assimilates to grain formation (Figure 1). This pattern of accumulation and allocation warrants further investigation in oats. Similar patterns have been reported in maize, where SG materials maintain photosynthetic activity for a longer duration during grain filling, thereby enhancing yield potential across different environments [25]. However, other studies have reported that changes in nitrogen remobilization may prevent extended photosynthesis in SG lines from translating into increased assimilate accumulation in grains [26].

The environmental conditions at the three experimental sites on the Qinghai–Tibet Plateau had a pronounced impact on the yield advantages of SG oats. At HZ, characterized by moderate temperatures (14–18 °C) and continuous rainfall (60–100 mm/month), SG lines achieved the highest grain yield and number of fertile spikelets (Figure 1B–D), suggesting that SG traits interact synergistically with environments offering sufficient moisture and favorable light–heat conditions during grain filling [27]. In contrast, the LD site, with higher temperatures (20–22 °C) and uneven rainfall, particularly heavy precipitation in September, posed challenges during the late growth stage. Despite these unfavorable conditions, SG lines maintained superior spike length and single-plant yield (Figure 1A,C), indicating resilience to short-term water stress. However, the benefits of SG traits were less pronounced at HY, the coolest and wettest site (12–15 °C; rainfall 90–120 mm/month), likely due to the reduced advantage of extended green leaf area in humid environments [28]. This observation is consistent with findings in other cereal crops, where SG benefits diminish under non-drought, moderate-temperature conditions.

SG lines exhibited significant increases in thousand-grain weight (TGW) across the different sites. At the LD site, TGW increased by 24.07%, potentially reflecting enhanced assimilate transport efficiency. An increase in grain width further supports this mechanism (Figure 2B). At HZ and HY, TGW also increased (+29.68% and +16.5%, respectively), likely due to an extended grain-filling duration, although further studies are required for confirmation. These results underscore the role of SG traits in enhancing the sink effect, particularly in environments that provide favorable conditions for grain filling.

Overall, these results highlight the production advantages of SG oat lines, demonstrating their superior yield potential across diverse environments. The SG lines enhance yield by maintaining photosynthetic activity for a longer duration during the grain-filling period, ensuring a sustained flow of assimilates to the developing grain. This mechanism is particularly beneficial in environments with optimal temperatures and adequate water availability during grain filling. Furthermore, SG traits provide strategic value for cultivating oats capable of adapting to increasingly unpredictable and changing climates, offering potential solutions for improving oat production under diverse ecological conditions.

### 3.2. Accumulation of Grain Nutritional Components in Stay-Green Oats

The SG oat lines exhibited pronounced advantages in grain nutritional quality traits, particularly in starch, protein, and water-soluble carbohydrate (WSC) contents (Figure 3). Notably, the most significant increases in starch content were observed at HZ and HY, both characterized by moderate temperatures and adequate rainfall during the grain-filling period—conditions conducive to sustained photosynthesis and carbohydrate accumulation. This advantage likely stems from prolonged photosynthetic activity in SG lines, which enhances assimilate availability for starch biosynthesis during grain development. These findings are consistent with previous studies in cereal crops, where the SG trait prolonged leaf functionality and improved carbohydrate deposition, thereby enhancing grain quality [25].

WSC content was significantly higher in SG lines at the HY site (Figure 3B). The cooler conditions at this site during the late growth stage likely promoted WSC (sucrose, glucose, and fructose) accumulation as an osmoprotectant. Such a response is commonly observed in oats under cold stress, where WSC stabilizes cell membranes and proteins, thereby enhancing stress resilience [29]. The SG lines likely supported this accumulation by prolonging photosynthesis and sugar metabolism throughout the grain-filling period, thereby improving the plant’s capacity to cope with low-temperature stress [28].

Protein content in SG lines was also significantly higher at both the HZ and HY sites (Figure 3C), where favorable environmental conditions likely reduced nitrogen volatilization losses and improved root function, thereby supporting efficient nitrogen acquisition during reproductive stages. This result aligns with previous studies indicating that SG traits enhance nitrogen use efficiency, thereby promoting protein synthesis in grains [10]. At the LD site, although protein content was generally higher, the SG-induced increase was less pronounced, possibly due to heavy rainfall at the end of the season, which hindered nitrogen remobilization efficiency [30].

Fat content exhibited a slight but significant increase at the LD and HY sites (Figure 3D). Although the increase was modest, higher lipid reserves can enhance energy supply during seed germination and early seedling establishment [31]. The increased lipid accumulation at HY, characterized by cooler temperatures and high rainfall, likely reflects an adaptive response to abiotic stress.

Overall, although genotype, environment, and their interactions influence the expression of SG advantages, SG lines consistently exhibit superior nutritional quality traits across most environments (e.g., HZ and HY), likely reflecting synergistic improvements in carbon–nitrogen metabolism and assimilate distribution. These results underscore the critical role of environmental screening in SG breeding, specifically in optimizing parental selection based on temperature and humidity conditions during the reproductive stage, as well as genotype characteristics. For instance, the QY3 genotype has consistently enhanced WSC content, while QY5 ensures stable protein content.

### 3.3. Seed Germination Quality of Stay-Green Lines

Under controlled laboratory conditions, this study demonstrated that the SG trait significantly enhanced seed germination and early seedling establishment in oats across multiple sites on the Qinghai–Tibet Plateau. This enhancement primarily resulted from genotype × environment interactions occurring during seed development. At the HZ site, SG lines (e.g., LN_SG) exhibited superior germination performance, with a 93.47% increase in thousand-grain weight (TGW) and significant enhancements in starch and protein contents, thereby supplying greater energy and nutrient reserves. In contrast, seeds from the LD and HY sites exhibited reduced germination uniformity, likely due to temperature fluctuations and uneven precipitation. Nevertheless, SG lines still achieved TGW increases of 24.07% at LD and 16.5% at HY, accompanied by improvements in nutritional quality.

The SG group had consistently enhanced germination energy and seedling vigor, though the degree varied by genotype. QY3_SG maintained higher germination energy consistently across all environments, whereas QY5_SG exhibited significantly improved germination percentage, specifically at LD and HZ. LN_SG demonstrated superior seedling vigor under the more stressful HY conditions, likely attributable to prolonged photosynthesis that enhanced carbon and nitrogen assimilation and nutrient accumulation [13] (e.g., increased protein content at HY).

The mechanisms underlying the improved seed and seedling vigor can be summarized as follows: (a) SG enhanced grain quality, evidenced by a 16.5–29.68% increase in TGW (up to 93.47% at HZ), reflecting greater dry matter accumulation [12]. (b) SG optimized nutrient reserves, with higher starch and protein contents at HZ and HY ensuring sufficient energy supply for germination [22].

In summary, the SG lines enhanced seed quality and early seedling establishment through genotype × environment interactions by increasing TGW and nutrient reserves. The stable performance of QY3_SG and the specific adaptability of LN_SG at HY offer valuable genetic resources for oat breeding. Prolonged photosynthetic activity underpins these advantages by enhancing assimilate supply during grain filling. However, further research is required to elucidate how environmental factors modulate the SG effect and to optimize its application in breeding programs.

### 3.4. Comprehensive Evaluation of Stay-Green Oat: Genotype × Environment Interactions, Stability, and Breeding Implications

This study employs a comprehensive evaluation system combined with multi-trait stability analysis to demonstrate the pivotal role of the SG trait in enhancing oats’ adaptability across diverse environments. LN_SG exhibited excellent performance at all three experimental sites, demonstrating broad environmental adaptability and establishing itself as a highly promising breeding candidate [32]. Moreover, the SG group consistently conferred enhanced trait stability, likely mediated by delayed senescence mechanisms, including prolonged photosynthetic duration and improved nutrient retention. The QY5_SG genotype exemplifies this trait, exhibiting significant enhancements in seedling vigor parameters (notably root length and seedling length) while maintaining superior performance in yield and quality traits.

This study identified significant three-way interactions among location, genotype, and group (CK/SG) influencing nutrient composition. This finding underscores the necessity of integrating multiple factors in breeding programs, as exclusive focus on nutrient composition may inadvertently reflect influences beyond genotype. Therefore, breeding strategies targeting specific nutritional profiles must account for the complex interactions among these factors. In contrast, yield-related traits and grain morphology exhibited minimal three-way interaction effects (*p* > 0.05). Similarly, seed germination traits—such as germination energy, germination index, and seedling percentage—were minimally influenced by the three-way interaction (*p* > 0.05). These findings suggest that cereal grain analyses should adopt a cautious and comprehensive approach. Incorporating germination assessments alongside yield and nutritional evaluations not only mitigates the confounding effects of environmental and interaction factors but also enables a more holistic evaluation of germplasm. This multidimensional evaluation strategy aligns with contemporary crop breeding priorities emphasizing trait stability and environmental adaptability [33].

Although certain traits were influenced by environment, genotype, and their interactions, LN_SG consistently maintained a significant overall adaptive advantage, outperforming other genotypes across all experimental sites. This indicates that, although influenced by environmental factors, the stay-green trait reliably confers advantages in yield, quality, and germination stability under variable conditions. This finding concurs with previously reported positive effects of the stay-green trait on stress tolerance and yield stability in other crop species [34].

To systematically assess genotype performance across environments, this study employed a tiered analytical approach to improve clarity and robustness. Initial trait stability and genotype–environment responses were evaluated using coefficient of variation (CV) and interaction plots, offering preliminary insights. Due to complex multi-way interactions, these alone were insufficiently clear. Thus, the AMMI model was used for dimensionality reduction and intuitive visualization of adaptability and stability. Finally, TOPSIS integrated multiple traits into a single index to aid optimal genotype selection. This framework effectively identified broadly adaptable and stable genotypes like LN_SG and QY5_SG, demonstrating the benefit of combining complementary analyses for multi-trait, multi-environment evaluation.

In conclusion, this study presents a comprehensive evaluation framework to guide targeted improvement strategies for high-quality oat germplasm. This framework integrates complementary analyses to address multi-trait and multi-environment complexities, enhancing the robustness of genotype evaluation. To mitigate the confounding effects of multiple environments and their interactions, the evaluation framework should be as comprehensive as possible, avoiding overreliance on a limited set of traits such as yield, quality, or germination. However, given time and resource constraints, employing core trait assessments for preliminary screening of extensive germplasm collections remains a practical approach. Furthermore, incorporation of SG traits into breeding programs can significantly facilitate the achievement of high-yield and superior-quality breeding objectives in oats. It is noteworthy that the retention of green leaves and higher moisture content in stay-green genotypes at grain maturity may pose challenges for mechanical harvesting and potentially affect threshing efficiency. However, this trait simultaneously enhances the forage value of oat plants at harvest, making these genotypes particularly promising for dual-purpose use as both grain and high-quality fodder. Further research is needed to evaluate their performance and suitability under different harvesting regimes and end-use scenarios.

Although this study primarily relies on data from the 2024 three-site trials, Supplementary Materials include data from the 2023 HZ site. These data are consistent with the trends observed in 2024, showing that stay-green lines consistently demonstrated significant advantages in yield (Supplementary Table S1), nutritional quality traits (Supplementary Table S2), and seed germination indicators (Supplementary Table S3), thereby further confirming the superior performance of the stay-green trait across diverse environments.

### 3.5. Limitations and Future Perspectives

This study systematically evaluated the performance of stay-green (SG) oat genotypes across representative high-altitude environments of the Qinghai–Tibet Plateau. The results demonstrate that the SG trait enhances grain yield, nutritional quality, and seedling establishment through improved assimilate accumulation during grain filling. These findings provide valuable evidence supporting the application of SG traits in oat breeding under altitude-related environmental stress.

However, there are still some limitations. This study was conducted only in a single growing season. Although multi-point experiments revealed clear genotypic performance differences, the long-term stability of SG traits under more variable climatic conditions needs to be further verified by multi-year evaluation. In addition, the climate of the Qinghai–Tibet Plateau is cold. Although high temperatures have been seen occasionally in recent years, the study of extreme climate on oat growth should still be further studied in a larger oat production area. Therefore, future studies should be extended to low-altitude arid and high-temperature environments and include genotypes (such as QY3) that are more sensitive to environmental changes to further elucidate the adaptability and response patterns of SG traits.

## 4. Materials and Methods

### 4.1. Experiment Sites

**Figure 8:**
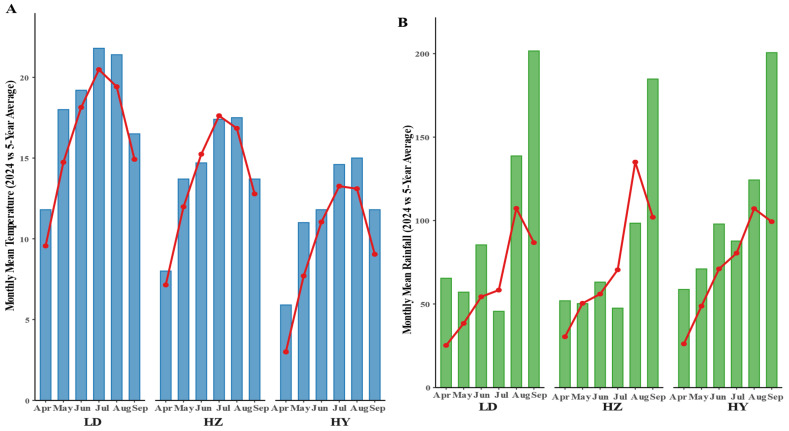
Comparison of monthly mean temperature and rainfall in 2024 and the 5-year average (2020–2024) across three experimental sites representing different environments. (**A**,**B**) present the monthly mean temperature and rainfall, respectively, from April to September at three experimental sites (LD, HZ, and HY) representing distinct environments. Bars indicate the observed values in 2024, while lines represent the 5-year average (2020–2024).

Field experiments were conducted in 2024 at three locations in Qinghai Province, China: Ledu District (LD), Huangzhong District (HZ), and Haiyan County (HY). LD is located at 102°09′~102°47′ E and 36°16′~36°40′ N, with an average elevation of 2183 m. The region has a typical plateau continental climate, with a mean annual temperature of 6.9 °C, annual precipitation of 335.4 mm, and annual evaporation of 1650 mm. HZ is located at 101°09′~101°54′ E and 36°13′~37°03′ N, with an average elevation of 2592 m. It has a plateau continental climate, with a mean annual temperature of 5.1 °C, precipitation of 510 mm, and evaporation of 1830 mm. HY is situated at 100°23′~101°20′ E and 36°44′~37°39′ N, at an elevation of 3156 m. The mean annual temperature is 0.5 °C, with 369.1 mm of precipitation and 1400 mm of evaporation. These locations represent diverse agroecological zones of the Qinghai–Tibet Plateau. The climatic data for the three experimental sites in 2024, as well as the multi-year averages, are presented in Figure 8. The data were obtained from the Qinghai Provincial Bureau of Statistics (http://tjj.qinghai.gov.cn/, accessed on 24 February 2025).

### 4.2. Experimental Design and Field Management

A randomized complete block split-plot design was employed, with location as the main plot factor and six oat materials as subplot treatments: LN_CK, LN_SG, QY3_CK, QY3_SG, QY5_CK, and QY5_SG. These materials were derived from three genotypic backgrounds (LN, QY3, and QY5), each of which included both a control (CK) line and a corresponding stay-green (SG) variant. As each genotype appears in both group levels, genotype and group were treated as independent (crossed) factors in the subsequent analysis. All materials were provided by the Qinghai Academy of Animal Science and Veterinary. LN_CK and LN_SG (medium maturity, grain type), QY3_CK and QY3_SG (late maturity, grain and feed type), QY5_CK and QY5_SG (medium and late maturity, grain and feed type) were included in the study. Among them, LN_CK and QY3_CK are officially registered and widely planted varieties on the Qinghai–Tibet Plateau. QY5-CK, LN-SG, QY3_SG and QY5_SG are new lines with stable phenotypes after long-term field selection by their respective parent materials. LN-SG, QY3_SG and QY5_SG are new oat lines with stable stay-green phenotype. Stay-green genotypes were identified visually: at physiological grain maturity, when traditional cultivars had completed leaf and stem senescence, selected lines still exhibited visibly green foliage. This phenotypic criterion was consistently applied across all plots. Throughout the manuscript, the term group (CK/SG) refers to the genotype grouping based on stay-green expression: control (CK) vs. stay-green (SG) variants.

In each location, three randomized complete blocks were established. Each block contained six subplots (one per material), with subplot sizes of 15 m^2^ (3 m × 5 m), 20 cm row spacing, and 1 m between blocks. Additionally, 150 kg/ha of diammonium phosphate and 75 kg/ha of urea were applied as basal fertilizers before sowing, and manual weeding was performed three times. The planting density was 6.75 × 10^6^ plants/ha.

### 4.3. Plant Harvest and Agronomic Traits Measurement

At the physiological maturity stage (PM), a total of 15 representative plants were randomly selected from each genotype and group (CK/SG) combination (including both the stay-green lines and the controls; 5 plants per plot, 3 plots per combination) for the measurement of spike-related yield traits and grain traits, including: spike length (SL, cm), from spike base to spike tip; number of fertile spikelets (FSN); yield per plant (SPY, g); plot yield (PY, g); thousand-grain weight (TGW, g); grain length (GL, mm), and grain width (GW, mm). In addition, a 1 m^2^ area was randomly selected in each plot to collect oat grains. The sample was inactivated at 105 °C for 30 min to terminate the enzyme activity, then dried to constant weight at 65 °C, ground, and sieved through a 40-mesh sieve for nutritional analysis. The oats used in this study are spring-type oats, as indicated by the sowing dates in spring 2024. Sowing and harvesting dates were as follows: LD, 7 April to 18 August 2024; HZ, 21 April to 1 September 2024; HY, 9 May to 19 September 2024.

### 4.4. Determination of Grain Nutritional Quality

Starch and water-soluble carbohydrates contents were determined using the anthrone method [35]. Crude protein content was measured via the semi-micro Kjeldahl method [36], and crude fat was determined using Soxhlet extraction [37].

### 4.5. Quality Evaluation of Oat Seed Germination

Seeds were surface-sterilized with 5% NaClO for 10 min, rinsed 4–5 times with distilled water, and dried using filter paper. For germination, 50 seeds were evenly placed in 11 × 11 cm germination boxes lined with three layers of filter paper, with four replicates per material. Each box received 15 mL of distilled water and was pre-chilled at 4 °C for 5 days to break dormancy. Germination was conducted under a 16 h light/8 h dark cycle at 5000 lux in a growth chamber (ZRQ-250, Zhetu Scientific Instrument Co., Ltd., Shanghai, China) during November–December 2024. Germination was defined as radicle emergence ≥ 2 mm. On day 10, 10 normal seedlings were randomly selected per box for measurement of seedling length (SL), root length (RL), seedling weight (SW), and root weight (RW) using a measuring tape and electronic balance (AX224ZH/E, Precision Instrument Co., Ltd., Shanghai, China).

Additionally, the germination energy (GE), germination percentage (GP), germination index (GI), seed vigor index (VI), seedling percentage (SP), and seedling vigor index (SVI) were calculated.

$$\begin{array}{c} GE=(N_{4}/N)\times 100\%\\ GP=(N_{10}/N)\times 100\%\\ GI=\Sigma (N_{t}/D_{t})\\ VI=(\Sigma (N_{t}/D_{t}))\times (SW+RW)\\ SP=(G_{10}/N)\times 100\%\\ SVI=(SL+RL)\times SP \end{array}$$

In the above equations, *N*_4_ and *N*_10_ represent the number of germinated seeds counted on day 4 and day 10, respectively; *G*_10_ is the number of normal seedlings counted on day 10; *N* is the total number of seeds tested; *N*_t_ is the number of germinated seeds counted on day t; and *D*_t_ is the germination day.

### 4.6. Data Analysis

Data were processed with Microsoft Excel 2021. Normality and homogeneity of variance were tested with IBM SPSS Statistics 27. For each trait, a three-way analysis of variance (ANOVA) was conducted using a generalized linear model (GLM) in R 4.5.0, with location (LD, HZ, HY), genotype (LN, QY3, QY5), and group (CK, SG) as fixed factors, including all main effects and interactions. The “Anova()” function from the “car” package with Type II sum of squares was applied to handle unbalanced data. Multiple comparisons among group means were performed using the Games–Howell test (*p* < 0.05).

Stability and adaptability across environments were initially assessed by calculating the coefficient of variation (CV) for each genotype based on data from all locations, providing a preliminary measure of performance variability. More comprehensive analysis of adaptability and stability was conducted using the additive main effects and multiplicative interaction (AMMI) model, which combines analysis of variance (ANOVA) and principal component analysis (PCA). The AMMI analysis was performed in R, with AMMI1 biplots generated using the tidyverse and ggplot2 packages.

Multi-trait evaluation was conducted using the Technique for Order Preference by Similarity to Ideal Solution (TOPSIS) method implemented with the “plyr” package in R. TOPSIS calculates the Euclidean distance between each genotype and both the ideal (best) and negative ideal (worst) solutions, generating a comprehensive evaluation score for genotype ranking. This method effectively integrates multiple traits into a single index and has been widely used in crop evaluation studies [38,39].

All figures were generated in R 4.5.0 and further refined using Microsoft PowerPoint.

## 5. Conclusions

The stay-green (SG) lines showed significant yield advantages, nutritional quality advantages and seedling establishment advantages in different ecological environments on the Qinghai–Tibet Plateau. They outperformed controls in key yield-related traits, such as spike length, fertile spikelets per plant, yield per plant, and thousand-grain weight. SG lines also exhibited improved starch content and, in some environments, higher protein accumulation. Additionally, they showed superior germination energy, germination rate, vigor indices, and seedling morphological traits, highlighting a consistent advantage from seed germination to early seedling growth. Adaptability analyses revealed that SG lines, particularly ‘LN_SG,’ exhibited the greatest stability and environmental adaptability. ‘LN_SG’ combined stable yield, superior nutritional quality, and broad environmental adaptability, making it a promising candidate material for breeding programs aimed at enhancing oat productivity in variable environments.

## Data Availability

Data are contained within the article and Supplementary Materials.

## Supplementary Materials

The following supplementary data are available at https://www.mdpi.com/article/10.3390/plants14162500/s1. Supplementary Table S1: Analysis of yield and grain traits of HZ in 2023. Supplementary Table S2: Nutrient composition analysis of HZ grain in 2023. Supplementary Table S3: Analysis of germination-related indicators of HZ harvested seeds in 2023.

## References

[1] Mao L., Zhang H., Yang Z., Li Y., Shen Y. (2025). Site-specific effects of fertilizer on hay and grain yields of oats: Evidence from large-scale field experiments. J. Sci. Food Agric..

[2] Paudel D., Dhungana B., Caffe M., Krishnan P. (2021). A Review of Health-Beneficial Properties of Oats. Foods.

[3] Tamiru M., Alkhtib A., Belachew B., Demeke S., Worku Z., Wamatu J., Burton E. (2023). Oat–Field Pea Intercropping for Sustainable Oat Production: Effect on Yield, Nutritive Value and Environmental Impact. Sustainability.

[4] Thomas H., Ougham H. (2014). The stay-green trait. J. Exp. Bot..

[5] Kamal N.M., Gorafi Y.S.A., Abdelrahman M., Abdellatef E., Tsujimoto H. (2019). Stay-Green Trait: A Prospective Approach for Yield Potential, and Drought and Heat Stress Adaptation in Globally Important Cereals. Int. J. Mol. Sci..

[6] Peng Y.Y., Liao L.L., Liu S., Nie M.M., Li J., Zhang L.D., Ma J.F., Chen Z.C. (2019). Magnesium Deficiency Triggers SGR–Mediated Chlorophyll Degradation for Magnesium Remobilization. Plant Physiol..

[7] Shin D., Lee S., Kim T.-H., Lee J.-H., Park J., Lee J., Lee J.Y., Cho L.-H., Choi J.Y., Lee W. (2020). Natural variations at the Stay-Green gene promoter control lifespan and yield in rice cultivars. Nat. Commun..

[8] Kumar R., Harikrishna, Barman D., Ghimire O.P., Gurumurthy S., Singh P.K., Chinnusamy V., Padaria J.C., Arora A. (2022). Stay-green trait serves as yield stability attribute under combined heat and drought stress in wheat (*Triticum aestivum* L.). Plant Growth Regul..

[9] Ali A., Ullah Z., Sher H., Abbas Z., Rasheed A. (2023). Water stress effects on stay green and chlorophyll fluorescence with focus on yield characteristics of diverse bread wheats. Planta.

[10] Liu Z., Hu C., Wang Y., Sha Y., Hao Z., Chen F., Yuan L., Mi G. (2021). Nitrogen allocation and remobilization contributing to low-nitrogen tolerance in stay-green maize. Field Crops Res..

[11] Borrell A.K., Wong A.C.S., George-Jaeggli B., van Oosterom E.J., Mace E.S., Godwin I.D., Liu G., Mullet J.E., Klein P.E., Hammer G.L. (2022). Genetic modification of PIN genes induces causal mechanisms of stay-green drought adaptation phenotype. J. Exp. Bot..

[12] Richards R.A. (2000). Selectable traits to increase crop photosynthesis and yield of grain crops. J. Exp. Bot..

[13] Malakondaiah A.C., Arora A., Krishna H., Taria S., Kumar S., Devate N.B., Padaria J.C., Kousalya S., Patil S.P., Singh P.K. (2025). Genome-wide association mapping for stay-green and stem reserve mobilization traits in wheat (*Triticum aestivum* L.) under combined heat and drought stress. Protoplasma.

[14] Li Y., Tao F., Hao Y., Tong J., Xiao Y., He Z., Reynolds M. (2022). Wheat traits and the associated loci conferring radiation use efficiency. Plant J..

[15] Zhang L.-l., Zhou X.-l., Fan Y., Fu J., Hou P., Yang H.-l., Qi H. (2019). Post-silking nitrogen accumulation and remobilization are associated with green leaf persistence and plant density in maize. J. Integr. Agric..

[16] Wang P., Hou S., Wen H., Wang Q., Li G. (2021). Chlorophyll retention caused by STAY-GREEN (SGR) gene mutation enhances photosynthetic efficiency and yield in soybean hybrid Z1. Photosynthetica.

[17] Shao H., Dongfeng S., Wenjun S., Xiangben B., Yachao C., Wei R., Fanjun C., Mi G. (2021). The impact of high plant density on dry matter remobilization and stalk lodging in maize genotypes with a different stay-green degree. Arch. Agron. Soil Sci..

[18] Sadras V.O., Mahadevan M., Zwer P.K. (2019). Stay-green associates with low water soluble carbohydrates at flowering in oat. Field Crops Res..

[19] Mel R., Malalgoda M. (2022). Oat protein as a novel protein ingredient: Structure, functionality, and factors impacting utilization. Cereal Chem..

[20] Kouřimská L., Sabolová M., Horčička P., Rys S., Božik M. (2018). Lipid content, fatty acid profile, and nutritional value of new oat cultivars. J. Cereal Sci..

[21] Zhu F. (2017). Structures, properties, modifications, and uses of oat starch. Food Chem..

[22] Basu S., Groot S.P. (2023). Seed vigour and invigoration. Seed Science and Technology: Biology, Production, Quality.

[23] García-Parra M., Roa-Acosta D., Stechauner-Rohringer R., García-Molano F., Bazile D., Plazas-Leguizamón N. (2020). Effect of temperature on the growth and development of quinoa plants (*Chenopodium quinoa* Willd.): A review on a global scale. Sylwan.

[24] De Santis M.A., Soccio M., Laus M.N., Flagella Z. (2021). Influence of Drought and Salt Stress on Durum Wheat Grain Quality and Composition: A Review. Plants.

[25] Chibane N., Caicedo M., Martinez S., Marcet P., Revilla P., Ordás B. (2021). Relationship between Delayed Leaf Senescence (Stay-Green) and Agronomic and Physiological Characters in Maize (*Zea mays* L.). Agronomy.

[26] Swanckaert J., Pannecoucque J., Van Waes J., Steppe K., Van Labeke M.C., Reheul D. (2017). Stay-green characterization in Belgian forage maize. J. Agric. Sci..

[27] Mahadevan M., Calderini D.F., Zwer P.K., Sadras V.O. (2016). The critical period for yield determination in oat (*Avena sativa* L.). Field Crops Res..

[28] Jinqiu Y., Bing L., Tingting S., Jinglei H., Zelai K., Lu L., Wenhua H., Tao H., Xinyu H., Zengqing L. (2021). Integrated Physiological and Transcriptomic Analyses Responses to Altitude Stress in Oat (*Avena sativa* L.). Front. Genet..

[29] Batool M., El-Badri A.M., Wang C., Mohamed I.A.A., Wang Z., Khatab A., Bashir F., Xu Z., Wang J., Kuai J. (2022). The role of storage reserves and their mobilization during seed germination under drought stress conditions of rapeseed cultivars with high and low oli contents. Crop Environ..

[30] Wang P., Fu C., Wang L., Yan T. (2022). Delayed autumnal leaf senescence following nutrient fertilization results in altered nitrogen resorption. Tree Physiol..

[31] Kandasamy S., Weerasuriya N., Gritsiouk D., Patterson G., Saldias S., Ali S., Lazarovits G. (2020). Size Variability in Seed Lot Impact Seed Nutritional Balance, Seedling Vigor, Microbial Composition and Plant Performance of Common Corn Hybrids. Agronomy.

[32] Liebsch D., Juvany M., Li Z., Wang H.L., Ziolkowska A., Chrobok D., Boussardon C., Wen X., Law S.R., Janečková H. (2022). Metabolic control of arginine and ornithine levels paces the progression of leaf senescence. Plant Physiol..

[33] Lee S., Lee H., Lee C., Ha S., Park H., Lee S., Kwon Y., Jeung J., Mo Y. (2023). Multi-Environment Trials and Stability Analysis for Yield-Related Traits of Commercial Rice Cultivars. Agriculture.

[34] Rehman H.U., Tariq A., Ashraf I., Ahmed M., Muscolo A., Basra S.M.A., Reynolds M. (2021). Evaluation of Physiological and Morphological Traits for Improving Spring Wheat Adaptation to Terminal Heat Stress. Plants.

[35] Hansen J., Møller I. (1975). Percolation of starch and soluble carbohydrates from plant tissue for quantitative determination with anthrone. Anal. Biochem..

[36] Nowak V., Du J., Charrondière U.R. (2016). Assessment of the nutritional composition of quinoa (*Chenopodium quinoa* Willd.). Food Chem..

[37] Srigley C.T., Mossoba M.M. (2016). Current Analytical Techniques for Food Lipids. Food Safety.

[38] Tzeng G.-H., Huang J.-J. (2011). Multiple Attribute Decision Making: Methods and Applications.

[39] Yu J., Bai X., Zhang K., Feng L., Yu Z., Jiao X., Guo Y. (2024). Assessment of Breeding Potential of Foxtail Millet Varieties Using a TOPSIS Model Constructed Based on Distinctness, Uniformity, and Stability Test Characteristics. Plants.

