# Supplementary material for: Stay-Green Trait Enhances Grain Yield, Nutritional Quality, and Seed Germination Ability in Oat (Avena sativa L.) on the Qinghai–Tibet Plateau
Source: Plants (Basel). 2025 Aug 12;14(16):2500. doi: 10.3390/plants14162500 (PMC12389223; doi:10.3390/plants14162500)
Supplement: Supplementary file 1 [file plants-14-02500-s001.zip › plants-3711358-Supplementary Tables.pdf]

Supplementary Table S1: Analysis of yield and grain traits of HZ in 2023.

Indicator	Genotype	CK	SG	Significance[1]
Spike length (cm)	LN	16.41 ± 0.40 E/c	17.38 ± 0.65 D/c	ns
	QY3	19.18 ± 0.67 C/b	20.87 ± 0.57 B/b	ns
	QY5	22.23 ± 0.59 A/a	24.04 ± 1.02 A/a	ns
	**Mean**	**19.27 ± 0.54**	**20.76 ± 0.66**	**ns**
Fertile spikelet per plant (count per plant)	LN	46.00 ± 3.25 B/a	47.20 ± 3.43 A/a	ns
	QY3	34.10 ± 2.05 C/b	42.50 ± 1.87 B/a	**
	QY5	34.08 ± 2.61 B/b	35.30 ± 2.32 B/b	ns
	**Mean**	**38.47 ± 1.75**	**41.27 ± 1.82**	**ns**
Single plant grain yield (g)	LN	2.31 ± 0.17 D/b	2.46 ± 0.23 C/b	ns
	QY3	2.61 ± 0.18 B/a	3.18 ± 0.18 A/a	*
	QY5	1.93 ± 0.12 D/c	1.81 ± 0.16 E/c	ns
	**Mean**	**2.28 ± 0.10**	**2.49 ± 0.15**	**ns**
Plot yield (g)	LN	1778.43 ± 224.34 B/a	1875.66 ± 105.99 B/a	ns
	QY3	2221.77 ± 232.24 B/a	2400.12 ± 11.68 A/a	ns
	QY5	1365.67 ± 103.90 C/a	1587.34 ± 201.72 B/a	ns
	Mean	1788.63 ± 157.68	1954.37 ± 135.97	ns
Grain length (mm)	LN	13.45 ± 0.29 A/a	13.11 ± 0.29 B/b	ns
	QY3	12.34 ± 0.15 C/c	13.29 ± 0.27 B/a	**
	QY5	12.34 ± 0.28 B/b	12.50 ± 0.10 C/c	ns
	**Mean**	**12.77 ± 0.17**	**12.91 ± 0.15**	**ns**
Grain width (mm)	LN	2.74 ± 0.03 B/b	2.87 ± 0.08 B/b	ns
	QY3	3.30 ± 0.02 A/a	3.36 ± 0.07 A/a	ns
	QY5	2.74 ± 0.04 B/b	2.93 ± 0.05 B/b	ns
	**Mean**	**2.93 ± 0.05**	**2.96 ± 0.06**	**ns**
Thousand grain weight (g)	LN	32.93 ± 0.42 D/b	41.93 ± 0.73 B/b	***
	QY3	41.23 ± 0.45 C/a	44.79 ± 0.60 A/a	***
	QY5	34.89 ± 0.82 D/b	41.50 ± 0.43 C/b	***
	**Mean**	**36.35 ± 0.74**	**42.74 ± 0.43**	***

Values are presented as Mean ± SE. Different uppercase letters indicate significant differences among all 6 materials ($p < 0.05$). Different lowercase letters indicate significant differences among genotypes within the same phenotype ($p < 0.05$).
[1]Compares CK vs SG within the same genotype. ns: $p > 0.05$; *: $p \leq 0.05$; **: $p \leq 0.01$; ***: $p \leq 0.001$.

Supplementary Table S2: Nutrient composition analysis of HZ grain in 2023.

Indicator	Genotype	CK	SG	Significance[1]
Protein (% DW)	LN	9.06 ± 0.06 F/b	11.22 ± 0.04 C/b	***
	QY3	9.40 ± 0.13 E/b	9.54 ± 0.03 D/c	ns
	QY5	13.10 ± 0.01 B/a	13.54 ± 0.03 A/a	**
	**Mean**	**10.52 ± 0.65**	**11.43 ± 0.58**	**ns**
Fat (% DW)	LN	5.15 ± 0.01 C/a	5.41 ± 0.01 A/a	***
	QY3	4.59 ± 0.00 D/b	5.25 ± 0.01 B/b	***
	QY5	4.56 ± 0.01 E/b	4.54 ± 0.01 F/c	ns
	**Mean**	**4.77 ± 0.10**	**5.07 ± 0.13**	**ns**
Starch (% DW)	LN	22.88 ± 0.09 C/b	23.98 ± 0.03 A/a	**
	QY3	23.50 ± 0.04 B/a	22.01 ± 0.06 E/b	***
	QY5	21.60 ± 0.01 F/c	22.22 ± 0.05 D/b	**
	**Mean**	**22.66 ± 0.28**	**22.74 ± 0.31**	**ns**
Water soluble carbohydrates (% DW)	LN	26.79 ± 0.20 B/b	26.87 ± 0.14 B/b	ns
	QY3	29.44 ± 0.11 A/a	30.09 ± 0.17 A/a	*
	QY5	23.64 ± 0.15 C/c	23.49 ± 0.13 C/c	ns
	**Mean**	**26.62 ± 0.84**	**26.82 ± 0.95**	**ns**

Values are presented as Mean ± SE. Different uppercase letters indicate significant differences among all 6 materials ($p < 0.05$). Different lowercase letters indicate significant differences among genotypes within the same phenotype ($p < 0.05$).
[1]Compares CK vs SG within the same genotype. ns: $p>0.05$; *: $p≤0.05$; **: $p≤0.01$; ***: $p≤0.001$.

Supplementary Table S3: Analysis of germination related indicators of HZ harvested seeds in 2023.

Indicator	Genotype	CK	SG	Significance[1]
Germination energy	LN	92.67 ± 0.67 C/a	98.00 ± 0.00 B/a	ns
	QY3	92.67 ± 1.76 B/a	96.67 ± 1.76 B/a	ns
	QY5	94.67 ± 0.67 B/a	98.67 ± 0.67 A/a	*
	**Mean**	**93.33 ± 0.67**	**97.78 ± 0.62**	***
Germination percentage (%)	LN	95.33 ± 1.28 B/a	98.67 ± 0.67 B/b	ns
	QY3	98.67 ± 1.23 B/a	96.00 ± 0.06 C/c	ns
	QY5	97.00 ± 1.00 B/a	99.63 ± 0.37 A/a	ns
	**Mean**	**97.00 ± 0.76**	**98.10 ± 0.59**	**ns**
Germination index	LN	70.66 ± 0.90 A/a	69.35 ± 1.17 A/a	ns
	QY3	65.76 ± 1.26 A/a	69.63 ± 1.34 A/a	ns
	QY5	68.03 ± 0.10 A/a	70.29 ± 0.49 A/a	*
	**Mean**	**68.15 ± 0.84**	**69.76 ± 0.55**	**ns**
Seedling percentage (%)	LN	95.33 ± 1.33 A/a	98.67 ± 0.67 A/a	ns
	QY3	98.67 ± 1.33 A/a	96.00 ± 1.15 A/a	ns
	QY5	97.00 ± 1.00 A/a	99.20 ± 0.80 A/a	ns
	**Mean**	**97.00 ± 0.78**	**97.96 ± 0.67**	**ns**
Seed vigor index	LN	8.62 ± 0.11 A/a	8.46 ± 0.14 A/a	ns
	QY3	8.02 ± 0.15 A/a	8.49 ± 0.16 A/a	ns
	QY5	8.29 ± 0.01 A/a	8.57 ± 0.06 A/a	*
	**Mean**	**8.31 ± 0.10**	**8.50 ± 0.07**	**ns**
Seedling vigor index	LN	20.83 ± 0.14 D/a	22.66 ± 0.32 B/b	*
	QY3	16.69 ± 0.23 E/b	22.57 ± 0.01 C/b	**
	QY5	21.37 ± 0.00 D/a	35.30 ± 0.24 A/a	ns
	**Mean**	**19.63 ± 0.74**	**26.84 ± 2.12**	**
Seedling length (cm)	LN	14.13 ± 0.38 D/a	14.17 ± 0.03 C/c	ns
	QY3	9.33 ± 0.17 E/c	15.53 ± 0.15 B/b	***
	QY5	12.07 ± 0.58 A/b	17.63 ± 1.79 A/a	ns
	**Mean**	**11.84 ± 0.72**	**15.78 ± 0.72**	**
Root length (cm)	LN	7.73 ± 1.13 A/a	8.37 ± 0.09 C/c	ns
	QY3	6.23 ± 0.68 A/a	9.77 ± 0.15 B/b	*
	QY5	7.17 ± 0.73 A/a	9.93 ± 0.58 A/a	*
	**Mean**	**7.04 ± 0.49**	**9.36 ± 0.30**	**

Seedling weight (mg)	LN	75.60 ± 11.72 A/a	109.00 ± 6.35 A/a	ns
	QY3	67.33 ± 8.69 A/a	104.00 ± 3.46 A/a	*
	QY5	89.33 ± 0.88 A/a	102.83 ± 11.61 A/a	ns
	**Mean**	**77.42 ± 5.30**	**105.28 ± 4.06**	***
Root weight (mg)	LN	40.17 ± 14.03 Aa	78.50 ± 2.02 Aa	ns
	QY3	61.00 ± 14.57 Aa	75.77 ± 5.34 Aa	ns
	QY5	89.33 ± 16.60 Aa	73.93 ± 2.56 Aa	ns
	**Mean**	**63.50 ± 10.38**	**76.07 ± 1.92**	**ns**

Values are presented as Mean ± SE. Different uppercase letters indicate significant differences among all 6 materials ($p < 0.05$). Different lowercase letters indicate significant differences among genotypes within the same phenotype ($p < 0.05$).
[1]Compares CK vs SG within the same genotype. ns: $p>0.05$; *: $p\leq0.05$; **: $p\leq0.01$; ***: $p\leq0.001$.
